# High-performance blob-based iterative three-dimensional reconstruction in electron tomography using multi-GPUs

**DOI:** 10.1186/1471-2105-13-S10-S4

**Published:** 2012-06-25

**Authors:** Xiaohua Wan, Fa Zhang, Qi Chu, Zhiyong Liu

**Affiliations:** 1 Institute of Computing Technology and Key Lab of Intelligent Information Processing, Beijing, China; 2 Graduate University, Chinese Academy of Sciences, Beijing, China

## Abstract

**Background:**

Three-dimensional (3D) reconstruction in electron tomography (ET) has emerged as a leading technique to elucidate the molecular structures of complex biological specimens. Blob-based iterative methods are advantageous reconstruction methods for 3D reconstruction in ET, but demand huge computational costs. Multiple graphic processing units (multi-GPUs) offer an affordable platform to meet these demands. However, a synchronous communication scheme between multi-GPUs leads to idle GPU time, and a weighted matrix involved in iterative methods cannot be loaded into GPUs especially for large images due to the limited available memory of GPUs.

**Results:**

In this paper we propose a multilevel parallel strategy combined with an asynchronous communication scheme and a blob-ELLR data structure to efficiently perform blob-based iterative reconstructions on multi-GPUs. The asynchronous communication scheme is used to minimize the idle GPU time so as to asynchronously overlap communications with computations. The blob-ELLR data structure only needs nearly 1/16 of the storage space in comparison with ELLPACK-R (ELLR) data structure and yields significant acceleration.

**Conclusions:**

Experimental results indicate that the multilevel parallel scheme combined with the asynchronous communication scheme and the blob-ELLR data structure allows efficient implementations of 3D reconstruction in ET on multi-GPUs.

## Background

Electron tomography (ET) combines electron microscopy (EM) and tomographic imaging to elucidate three-dimensional (3D) descriptions of complex biological structures at molecular resolution [1]. In ET, a series of projection images are taken with an electron microscope from a unique biological sample at different orientations around a single tilt axis [2]. From those projection images, the 3D structure of the sample can be obtained by means of tomographic reconstruction algorithms [3]. Weighted backprojection (WBP) is a standard reconstruction method in the field of 3D reconstruction in ET, due to its algorithmic simplicity and computational efficiency [4]. The major disadvantage of WBP, however, is that the results may be strongly affected by limited-angle data and noisy conditions [5]. Iterative methods, such as Simultaneous Iterative Reconstruction Technique (SIRT), are one of the main alternatives to WBP in 3D reconstruction in ET, owing to their good performance in handling incomplete, noisy data [6,7]. Furthermore, blob-based iterative methods show a better performance than voxel-based ones in the incomplete and noisy conditions [5]. However, they have not been extensively used due to their high computational cost [8]. Furthermore, the need for high resolution makes ET of complex biological specimens use large projection images, which also yields large reconstructed files and requires an extensive use of computational resources and considerable processing time [8,9].

3D reconstruction in ET demands huge computational costs and resources that derive from the computational complexity of the reconstruction algorithms and the size and number of the projection images involved. Traditionally, high-performance computing [8] has been used to address such computational requirements by means of parallel computing on supercomputers [9], large computer clusters [10] and multicore computers [11]. Recently, graphics processing units (GPUs) offer an attractive alternative platform to cope with the demands in ET in terms of the high peak performance, cost effectiveness, and the availability of user-friendly programming environments, e.g. NVIDIA CUDA [12,13]. Several advanced GPU acceleration frameworks have been proposed to allow 3D-ET reconstruction to be performed on the order of minutes [14,15]. However, these parallel reconstructions on GPUs only adopt traditional voxel basis functions which are less robust than blob basis functions under noisy situations. Some previous work focuses on the blob-based iterative reconstruction on a single GPU, which is still time-consuming [16,17]. Single GPU cannot meet the requirements of the computational resources and the memory storage of 3D-ET reconstructions with the size of the projection images increasing constantly (typically 2 k × 2 k or even 4 k × 4 k). The architectural notion of a CPU serviced by multi-GPUs is an efficient solution for parallel 3D-ET reconstruction due to increasing the power of computations and the storage of memory.

Achieving efficient blob-based iterative reconstructions on multi-GPUs is challenging: because of the overlapping nature of blobs, the use of blobs as basis functions needs the communication between multiple GPUs during the process of iterative reconstructions. CUDA provides a synchronous communication scheme to handle the communication between GPUs [18]. But the downside of the synchronous communication is that each GPU must stop and sit idle while data is exchanged. The idle sit of GPU is a waste of resources which has a negative impact on the performance of reconstructions on multi-GPUs. Furthermore, as data collection strategies and electron detectors improve, a sparse weight matrix involved in blob-based iterative reconstruction methods needs large memory storage. Due to the limited available memory, it is infeasible to store such a large sparse matrix for most GPUs. Computing the weight matrix on the fly is more efficient than storing the matrix in the previous GPU-based ET implementations [14]. But it could bring the redundant computations since the weighted matrix has to be computed twice at least in each iteration.

To address the problems discussed above, in this paper, we make the following contributions: first, we present a multilevel parallel strategy for blob-based iterative reconstructions in ET on multi-GPUs, which can significantly accelerate 3D reconstructions in ET. Second, we develop an asynchronous communication scheme on multi-GPUs to minimize idle GPU time by asynchronously overlapping communications with computations. Finally, a data structure named blob-ELLR adopting three symmetric optimization techniques is developed to significantly reduce the storage space of the weight matrix. It only needs nearly 1/16 of the storage space in comparison with ELLPACK-R (ELLR). Also, the blob-ELLR format can achieve optimal coalesced access to global memory, which is suitable for 3D-ET reconstructions on multi-GPUs. Furthermore, we implement all the above techniques on the two different platforms: a NVIDIA GeForce GTX295 and two NVIDIA Tesla C2050s respectively. Experimental results show that the parallel strategy greatly reduces memory requirements and exhibits a significant acceleration.

## Related background

In ET, the projection images are acquired from a specimen through the so-called single-axis tilt geometry. The specimen is tilted over a range, typically from -60° (or -70°) to +60° (or +70°) due to physical limitations of microscopes, with small tilt increments (1° or 2°). An image of the same object area is recorded at each tilt angle and then the 3D reconstruction of the specimen can be obtained from a set of projection images by means of blob-based iterative methods. In this section, we give a brief overview of blob-based iterative reconstruction methods, describe an iterative method called SIRT, and present a GPU computational model.

### Blob-based iterative reconstruction methods

Iterative methods are based on the series expansion approach [19] in which 3D volume *f *is represented as a linear combination of a limited set of known and fixed basis functions *b_j_*, with appropriate coefficients *x_j_*, i.e.

(1) $$f\left(r,\phi_{1},\phi_{2}\right)\approx \overset{N}{\underset{j=1}{\sum }}x_{j}b_{j}\left(r,\phi_{1},\phi_{2}\right),$$

where (*r, ϕ*_1_, *ϕ*_2_) is a spherical coordinate, and *N *is the total number of the unknown variables *x_j_*. In 3D reconstruction, the basis functions used to represent the object greatly influences the reconstructed results. During the 1990s, spherically symmetric volume elements (called blobs) have been thoroughly investigated, and turned to be more suitable for representing natural structures than the traditional voxels due to their overlapping nature [20]. Blobs are smooth functions with a maximum value at its center which gradually decays to zero at its extreme limits, adopting generalized Kaiser-Bessel (KB) window functions:

(2) $$b\left(r\right)=\left\{\begin{array}{cc} \frac{{\left(\sqrt{1-{\left(r/a\right)}^{2}}\right)}^{m}I_{m}\left(\alpha \sqrt{1-{\left(r/a\right)}^{2}}\right)}{I_{m}\left(\alpha \right)}, & 0\leq r\leq a\\ 0, & \text{otherwise} \end{array}\right)$$

where *I_m_*(·) denotes the modified Bessel function of the first kind of order *m, a *is the radius of the blob, *α *is a non-negative real number controlling the shape of the blob. The choice of the parameters *m, a*, and *α *will influence the quality of the blob-based reconstructions. The basis functions that developed in [21] are used for the choice of the parameters in our algorithm (i.e., *a *= 2, *m *= 2 and *α *= 3.6).

In 3D-ET, the model of the image formation process is expressed by the following linear system:

(3) $$p_{i}\approx \overset{N}{\underset{j=1}{\sum }}w_{ij}x_{j},1\leq i\leq M$$

where *p_i _*denotes the *ith *measured image of *f *and M = B*S is the dimension of *p*, with *B *being the number of projection angles and *S *the number of projection values per view. *w_ij _*is a weighting factor representing the contribution of the *jth *basis function to the *ith *projection. Under such a model, the element *w_ij _*can be calculated according to the projected procedure as follows:

(4) $$\begin{array}{c} w_{ij}=1-\left(rf_{ij}-\text{int}\left(rf_{ij}\right)\right),\\ rf_{ij}=projected\left(x_{j},\theta_{i}\right), \end{array}$$

where *rf_ij _*is the projected value of the pixel *x_j _*at an angle *θ_i_. W *is defined as a sparse matrix with *M *rows and *N *columns where *w_ij _*is the element of *W*. In general, the storage demand of the weighted matrix *W *rapidly increases as the size and the number of projection images increase. For example, when the size of images is 2 k × 2 k, the storage demand of the weighted matrix approaches to 3.5 GB. It is hard to store such a large matrix in the most GPUs due to the limited memory of GPUs.

Under those assumptions, the image reconstruction problem can be modelled as the inverse problem of estimating the *x_j_*'s from the *p_i_*'s by solving the system of linear equations given by Eq. (3). This problem is usually resolved by means of iterative methods.

### Simultaneous iterative reconstruction technique (SIRT)

SIRT is a kind of all simultaneous iterative methods to solve the linear system which appears in image reconstruction. All simultaneous iterative methods (such as SIRT [22], component averaging methods (CAV) [23]) utilize the projection in the all directions to refine the current reconstruction in each iteration so that they are well suited for parallel computing on GPUs. In our work, we adopt SIRT to perform parallel reconstruction on multi-GPUs.

Typically, SIRT begins with an initial *X^(0) ^*and repeats the iterative processes [24]. Initially, SIRT starts with an arbitrary approximation which may deviate from the true value far away. So the number of iterations until convergence may be large. In order to accelerate convergence, SIRT further adopts a back projection technique (BPT), a simple WBP, to estimate the first approximation *X^(0) ^*[25]. In iterations, the residuals, i.e. the differences between the actual projections *P *and the computed projections of the current approximation *X^(k) ^*(*k *is the iterative number), are computed and then *X^(k) ^*is updated by the backprojection of these discrepancies. Thus, the algorithm produces a sequence of N-dimensional column vectors *X^(k)^*. The SIRT algorithm is typically written by the following expression:

(5) $$\left\{\begin{array}{c} {x_{j}}^{\left(0\right)}=\frac{\sum_{i=1}^{M}w_{ij}p_{i}}{\sum_{i=1}^{M}w_{ij}},j=1,2,\ldots ,N\\ {x_{j}}^{\left(k+1\right)}={x_{j}}^{\left(k\right)}+\frac{1}{\sum_{i=1}^{M}w_{ij}}\overset{M}{\underset{i=1}{\sum }}\frac{w_{ij}\left(p_{i}-\sum_{h=1}^{N}w_{ih}{x_{h}}^{\left(k\right)}\right)}{\sum_{h=1}^{N}w_{ih}} \end{array}\right).$$

SIRT produces fairly smooth reconstruction results but requires for convergence a large number of iterations since SIRT adopts a global strategy: an approximation is updated simultaneously by all the projection images [24]. SIRT updates each *x_j _*only once per iteration, which means its updating strategy is pixel-by-pixel.

### GPU computation model

Our algorithm is based on NVIDIA GPU architecture and compute unified device architecture (CUDA) programming model. GPU is a massively multi-threaded data-parallel architecture. NVIDIA GPUs contain a scalable array of streaming multiprocessors (SMs) each of which contains scalar processors (SPs). On the old Tesla architecture of GPUs, there are 8 SPs per SM while a SM contains 32 SPs in the new Fermi architecture GPUs. All the SPs in the same SM execute the same instructions synchronously in a Single Instruction Multiple Thread (SIMT) fashion [18]. During execution, 32 threads from a continuous section are grouped into a warp, which is the scheduling unit on each SM.

NVIDIA provides the programming model and software environment of CUDA. CUDA is an extension to the C programming language. A CUDA program consists of a host program that runs on CPU and a kernel program that executes on GPU itself. The host program typically sets up data and transfers it to and from the GPU, while the kernel program processes that data. Kernel, as a program on GPUs, consists of thousands of threads. Threads have a three-level hierarchy: grid, block, thread. A grid is a set of blocks that execute a kernel, and each block consists of hundreds of threads. All threads within a block can share the same on-chip memory and can be synchronized at a barrier. Each block can only be assigned to and executed on one SM.

CUDA provides a synchronous communication scheme (i.e. cudaThreadSynchronize()) to handle the communication between GPUs. With the synchronous scheme, all of threads on GPUs must be blocked until the data communication has been completed. CUDA devices use several memory spaces including global, local, shared, texture, constant memory and registers. Of these different memory spaces, global memory is the most plentiful. Global memory loads and stores by a half warp (16 threads) are coalesced in as few as one transaction (or two transactions in the case of 128-bit words) when certain access requirements are met. Coalesced memory accesses deliver a much higher efficient bandwidth than non-coalesced accesses, thus greatly affecting the performance of bandwidth-bound programs.

## Methods

### Multilevel parallel strategy for blob-based iterative reconstruction

The processing time of 3D reconstruction with blob-based iterative methods is a major challenge in ET due to large reconstructed data volume. So parallel computing on multi-GPUs is becoming paramount to cope with the computational requirement. We present a multilevel parallel strategy for blob-based iterative reconstruction and implement it on the OpenMP-CUDA architecture.

#### Coarse-grained parallel scheme using OpenMP

In the first level of the multilevel parallel scheme, a coarse-grained parallelization is straightforward in line with the properties of ET reconstruction. The single-tilt axis geometry allows data decomposition into slabs of slices orthogonal to the tilt axis. For this decomposition, the number of slabs equals to the number of GPUs, and each GPU reconstructs its own slab. Consequently, the 3D reconstruction problem can be decomposed into a set of 3D slabs reconstruction sub-problems. However, the slabs are interdependent due to the overlapping nature of blobs. Therefore, each GPU has to receive a slab which is composed of its corresponding own slices and additional redundant slices reconstructed in neighbour slabs. The number of redundant slices depends on the blob extension. In a slab, the own slices are reconstructed by the corresponding GPU and require information provided by the redundant slices from the neighbour GPUs. During the process of 3D-ET reconstruction, each GPU has to communicate with other GPUs for the additional redundant slices.

**Figure 1:**
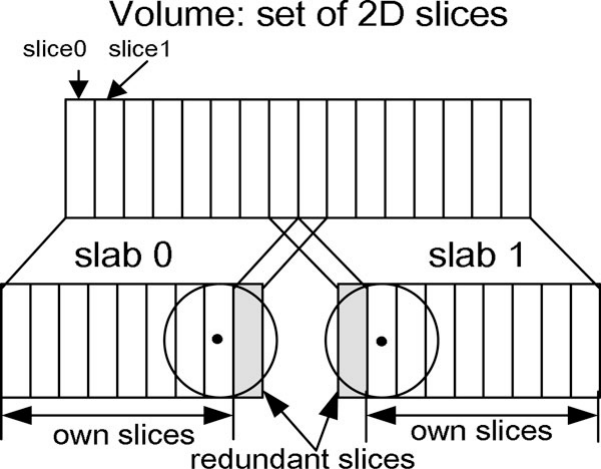
**Coarse-grained parallel scheme using blob**. 3D volume is decomposed into slabs of slices. The number of slabs equals the number of GPUs. Each GPU receives a slab. Each slab includes a set of own slices (in white) and an additional redundant slice (in gray) according to the shape of the blob.

We have implemented the 3D-ET reconstruction based on the architecture in which a CPU controls several GPUs and the GPUs share the memory. We adopt two GPUs in the different platforms to implement the blob-based reconstruction. Thus the first level parallel strategy makes use of two GPUs to perform the coarse-grained parallelization of the reconstruction. As shown in Figure 1, the 3D volume data is halved into two slabs, and each slab contains its own slices and a redundant slice. According to the shape of the blob adopted (the blob radius is 2 in our experiments), only one redundant slice is included in each slab. Each slab is assigned to and reconstructed on each individual GPU in parallel. A shared-memory parallel programming scheme (OpenMP) is employed to fork two threads to control the separated GPU. Each slab is reconstructed on each individual GPU by each parallel thread. Consequently, the partial results attained by GPUs are combined to complete the final result of the 3D reconstruction. Certainly, the parallel strategy can be applied on GPU clusters (e.g. Tesla-based cluster). In a GPU cluster, the number of slabs equals the number of GPUs for the decomposition described above.

#### Fine-grained parallel scheme using CUDA

In the second level of the multilevel parallel scheme, 3D reconstruction of one slab, as a fine-grained parallelization, is implemented on each GPU using CUDA. In the 3D reconstruction of a slab, the generic iterative process is described as follows:

• Initialization: compute the matrix *W *and make a initial value for *X^(0) ^*by BPT;

• Reprojection: estimate the computed projection data *P' *based on the current approximation *X*;

• Backprojection: backproject the discrepancy *ΔP *between the experimental and calculated projections, and refine the current approximation *X *by incorporating the weighted backprojection *ΔX*.

**Figure 2:**
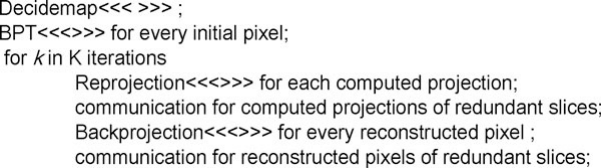
**Pseudo code for a slab reconstruction based on SIRT**.

Figure 2 shows the pseudo code for the 3D reconstruction by the aforementioned stages easily indentified in Eq. (5). We adopt the kernel called decidemap to compute the weighted matrix *W *and the kernel called BPT to estimate the initial value *X^(0) ^*in the initialization process. The interdependence among neighbour slabs due to the blob extension implies that, after computing either the reprojection or backprojection for a given slab, there must be a proper exchange of information between neighbour GPUs.

### Asynchronous communication scheme

As described above in the multilevel parallel scheme, there must be two communications between neighbour GPUs during one iterative reconstruction process. One is to exchange the computed projections of the redundant slices after the reprojection process. The other is to exchange the reconstructed pixels of the redundant slices after the backprojection process. CUDA provides a synchronous communication scheme (i.e. cudaThreadSynchronize()) to handle the communication between GPUs. With the synchronous communication scheme, GPUs must sit idle while data is exchange, which has a negative impact on the performance of the reconstruction in ET.

**Figure 3:**
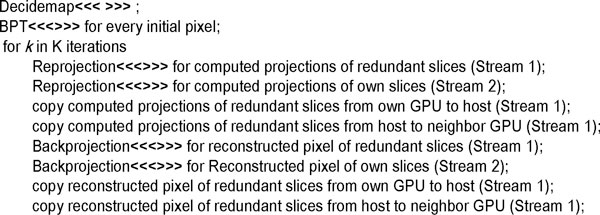
**Pseudo code for a slab reconstruction with the asynchronous communication scheme**.

In the 3D-ET reconstruction on clusters or supercomputers, the use of blobs as basis functions involves significant additional difficulties in the parallelization and makes substantial communications among the processors needed. In any parallelization project where communication between nodes is involved, latency hiding becomes an issue [9]. An effective strategy stands for overlapping communication and computation so as to keep the processor busy while waiting the communications to be completed [5]. In this work, a latency hiding strategy has been devised which has proven to be very efficient to deal with the communications [9]. To minimize the idle time on the GPUs, we also present a latency hiding strategy using an asynchronous communication scheme in which different streams are used to perform GPU execution and CPU-GPU memory access asynchronously. The communication scheme splits GPU execution and memory copies into separate streams. Execution in one stream can be performed at the same time as a memory copy from another. As shown in Figure 3, in one slab reconstruction, Reprojection of the redundant slices, memory copies and Backprojection of the redundant slices are performed in one stream. The executions (i.e. Reprojection and Backprojection) of the own slices are performed in the other stream. This can be extremely useful for reducing the idle time of GPUs.

### Blob-ELLR format with symmetric optimization techniques

In the parallel blob-based iterative reconstruction, another problem is the lack of memory on GPUs for the sparse weighted matrix. Recently, several data structures have been developed to store sparse matrices. Compressed row storage (CRS) is the most extended format to store the sparse matrix on CPUs [26]. ELLPACK can be considered as an approach to outperform CRS [27]. Vazquez et al. proposed and evaluated a variant of the ELLPACK format called ELLPACK-R (ELLR) [28]. ELLR has been proved to outperform the most efficient formats for storing the sparse matrix data structure on GPUs [29]. ELLR consists of two arrays, *A[] *and *I[] *of dimension N × MaxEntriesbyRows, and an additional N-dimensional integer array called *rl[] *is included in order to store the actual number of nonzeroes in each row [13,28]. With the size and number of projection images increasing, the memory demand of the sparse weighted matrix rapidly increases. The weighted matrix involved is too large to load into most of GPUs due to the limited available memory, even with the ELLR data structure.

**Figure 4:**
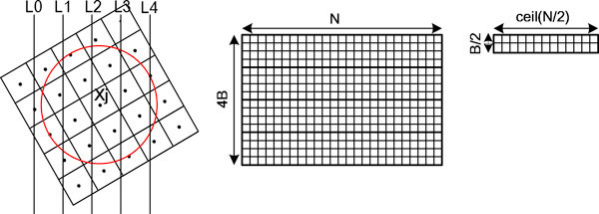
**Blob-ELLR format**. In the blob (the radius a = 2), a projected pixel X_j _contributes to four neighbour projection rays (L1, L2, L3 and L4) using only one view (left). The nonzeroes of W are stored in a 2D array A of dimension (4B) × N in blob-ELLR without symmetric techniques (middle). The symmetric optimization techniques are exploited to reduce the storage space of A to almost 1/16 of original size (right).

Recently, Vazquez et al. proposed a matrix approach and exploited several geometry related symmetry relationships to reduce the weighted matrix involved in WBP reconstruction method [13]. In our work, we present a data structure named blob-ELLR and also exploit several geometric related symmetry relationships to reduce the weighted matrix involved in iterative reconstruction methods. The blob-ELLR data structure decreases the memory requirement and then accelerates the speed of ET reconstruction on GPUs. Compared with a matrix approach to WBP using the original ELLR [13], our matrix blob-ELLR is adopted to store the weighted matrix *W *instead of the transpose of the one involved in the original ELLR. As shown in Figure 4, the maximum number of the rays related to each pixel is four on account of the radius of the blob (i.e., a = 2). To store the weighted matrix *W*, the blob-ELLR includes two 2D arrays: one float *A[] *to save the entries, and the other integer *I[] *to save the columns of every entry (see Figure 4 middle). Both arrays are of dimension *(4B) × N*, where *N *is the number of columns of *W *and *4B *is the maximum number of nonzeroes in the columns (*B *is the number of the projection angles). Because the percentage of zeros is low in the blob-ELLR, it is not necessary to store the actual number of nonzeroes in each column. Thus the additional integer array *rl[] *is not included in the blob-ELLR.

Although the blob-ELLR without symmetric techniques can reduce the storage of the sparse matrix *W*, the number of *(4B) × N *is rather large especially when the number of *N *increases rapidly. The optimization takes advantage of the symmetry relationships as follows:

• Symmetry 1

Assume that the *jth *column elements of the matrix *W *in each view are *w_0j_, w_1j_, w_2j _*and *w_3j_*. The relationship among the adjacent column elements is:

(6) $$w_{0j}=1+w_{1j};w_{2j}=1-w_{1j};w_{3j}=2-w_{1j}$$

So, only *w_1j _*is stored in the blob-ELLR, whereas the other elements are easily computed based on *w_1j _*. This scheme can reduce the storage spaces of *A *and *I *to 25%.

• Symmetry 2

Assume that a point (x, y) of a slice is projected to a point r (r = project(x, y, θ)) in the projection image corresponding to the tilt angle *θ *and project(x, y, θ) is shown as follows:

(7) project(x,y,θ)=xcosθ+ysinθ

It is easy to see that the point (-x,-y) of a slice is then projected to a point r1 (r1 = -r) in the same tilt angle *θ*. The weighted value of the point (-x,-y) can be computed according to that of the point (x, y). Therefore, it is not necessary to store the weighted value of almost a half of the points in the matrix *W *so that the space requirements for *A *and *I *are further reduced by nearly 50%.

• Symmetry 3

In general, the tilt angles used in ET are halved by 0°. Under the condition, a point (-x, y) with a tilt angle -θ is projected to a point r2 (r2 = -r). Therefore, the projection coefficients are shared with the projection of the point (x, y) with the tilt angle θ. This further reduces the storage spaces of *A *and *I *by nearly 50% again.

With the three symmetric optimizations mentioned above, the size of the storage for two arrays in the blob-ELLR is almost (B/2) × (N/2) reducing to nearly 1/16 of original size.

## Results and discussions

In order to evaluate the performance of the multilevel parallel strategy, the blob-based iterative reconstructions of the caveolae from the porcine aorta endothelial (PAE) cell have been performed [30]. Three different experimental datasets are used (denoted by small-sized, medium-sized, large-sized) with 56 images of 512 × 512 pixels, 112 images of 1024 × 1024 pixels, and 119 images of 2048 × 2048 pixels, to reconstruct tomograms of 512 × 512 × 190, 1024 × 1024 × 310 and 2048 × 2048 × 430 respectively. All the experiments are carried out on both GT200 and Fermi platforms. The details of the platforms are as follows. The GT200 machine consist of a 2.66 GHz Intel Xeon X5650, 24 GB RAM, and a NVIDIA GeForce GTX 295 card including two Tesla GPUs, each containing 30 SMs of 8 SPs (i.e. 240 SPs) at 1.2 GHz, 896 MB of memory. The Fermi machine is composed of the same CPU based on Intel Xeon X5650, and two NVIDIA Tesla C2050 cards. NVIDIA Tesla C2050 adopts the Fermi architecture and contains 14 SMs of 32 SPs (i.e. 448 SPs) at 1.15 GHz, 3 GB of memory. The two machines are both running on Redhat EL5 64-bit. For the comparison of the performance of multi-GPUs with CPU, we have performed the related serial program on the same CPU, i.e. Intel Xeon X5650, with a single core. To clearly evaluate the performance of the asynchronous communication scheme and the blob-ELLR data structure respectively, we have performed two sets of experiments. The details of the experiments are introduced below.

**Figure 5:**
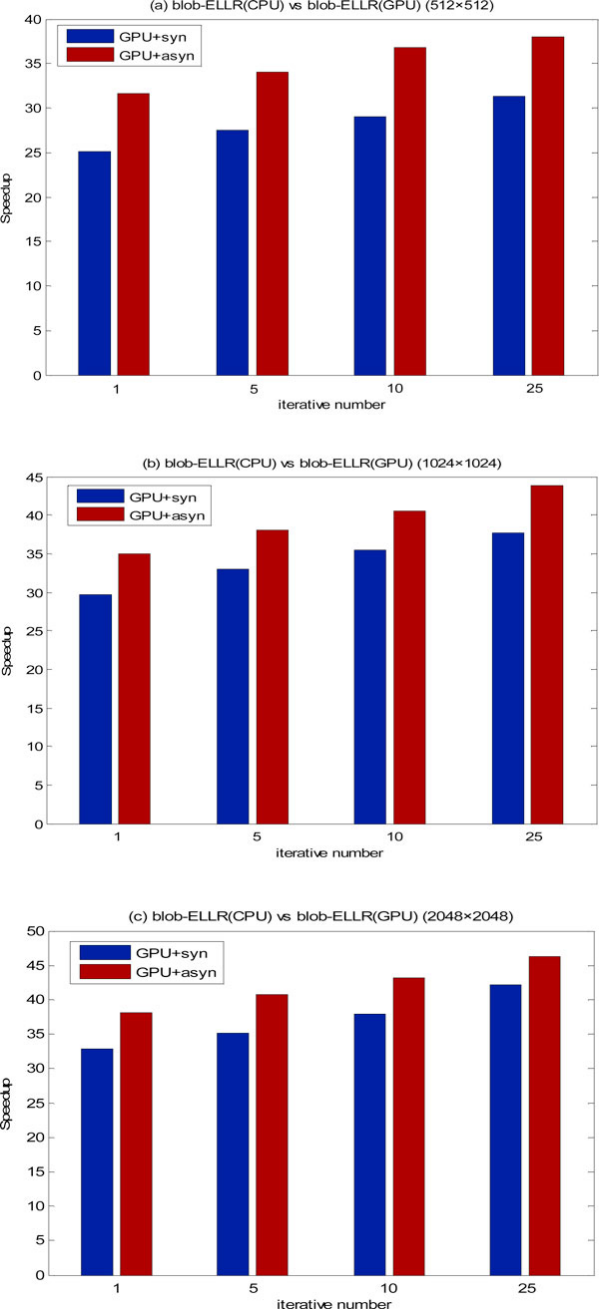
**Speedup factors of different iterations on the GTX295**. Speedup factors are showed by both implementations (with synchronous and asynchronous communication scheme respectively) over the reconstructions on the CPU. The results of the three datasets are shown in (a), (b) and (c), respectively.

In the first set of experiments, to estimate the performance of the asynchronous communication scheme, we have implemented and compared the blob-based iterative reconstruction of the three experimental datasets on the GTX295 and Tesla C2050s respectively. All the reconstructions adopt two methods separately: multi-GPUs with the synchronous communication scheme (named GPU+syn), and multi-GPUs with the asynchronous communication scheme (named GPU+asyn). In the experiments, the blob-ELLR developed in our work is used to storage the weighted matrix in the reconstruction. Figure 5 shows the speedups of the two communication schemes for different iterative number of reconstructions (i.e. 1, 5, 10, 25) using all the three datasets on the GTX295 vs. CPU. As shown in Figure 5, the speedups of GPU+asyn are larger than those of GPU+syn for the three experimental datasets. The asynchronous communication scheme exhibits excellent acceleration factors, reaching up to 35×, 40× and 45×, in the different datasets, respectively. The general rule is that the larger dataset and the more iterations performed, the larger speedup will be obtained using the asynchronous communication scheme.

**Figure 6:**
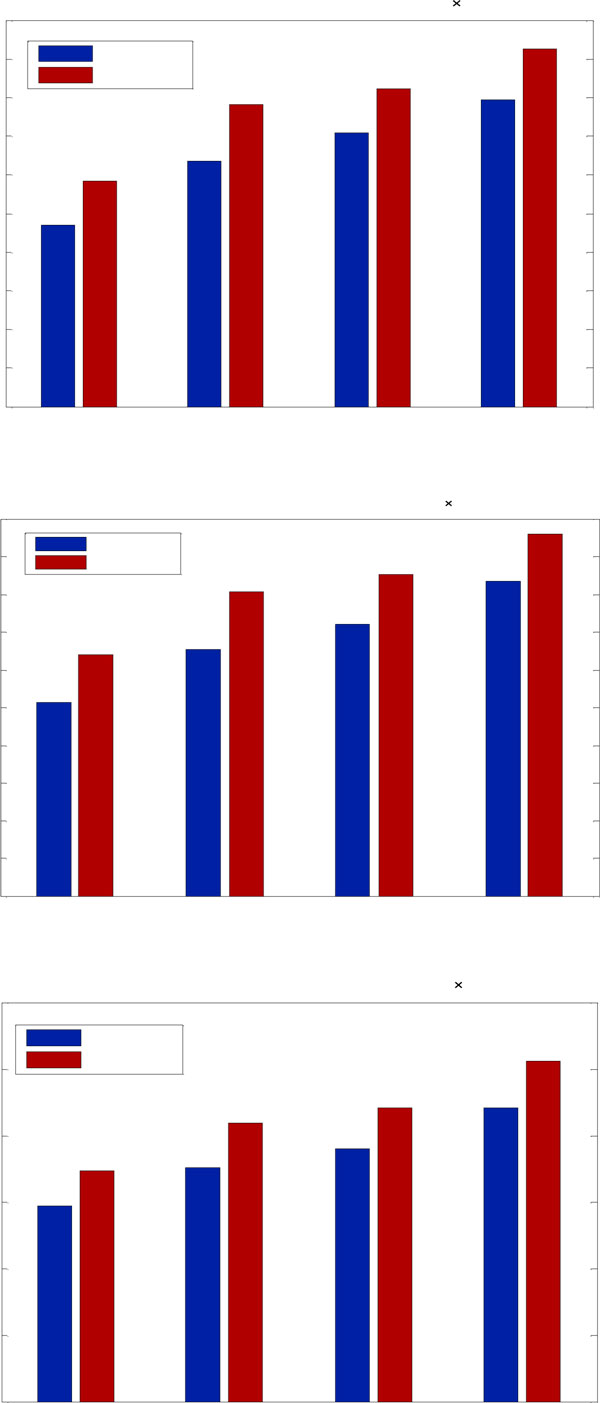
**Speedup factors of different iterations on the Tesla C2050**. Speedup factors are showed by both implementations (with synchronous and asynchronous communication scheme respectively) over the reconstructions on the CPU. The results of the three datasets are shown in (a), (b) and (c), respectively.

Figure 6 compares the speedups of different using the two communication schemes for all the three datasets on the Tesla C2050 vs. CPU. In this figure, we also observe that the speedups of GPU+asyn are larger than those of GPU+syn for the three experimental datasets. The asynchronous communication scheme exhibits excellent acceleration factors, reaching up to 90×, 95× and 100× for 25 iterations, in the different datasets respectively. The Tesla C2050s yields further speedups than the GTX295 mainly owing to the improvements of the Fermi architecture. In general, the asynchronous communication scheme provides the better performance than the synchronous scheme for the reason of asynchronous overlapping of communications and computations.

**Figure 7:**
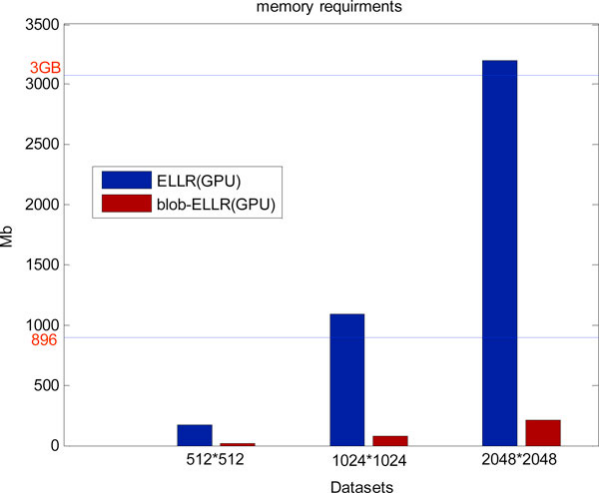
**Memory requirements of the different implementations for the datasets**. The limit memory in GTX295 is 896 MB and that in Tesla C2050 is 3 GB used in the work. The use of the blob-ELLR data structure reduces the memory demands, making most of the problems affordable.

In the second set of experiments, to compare the blob-ELLR data structure with other methods used for the weighted matrix, we have implemented the blob-based iterative reconstructions where the weighted matrices are computed on the fly (named standard matrix), pre-computed and stored with ELLR (named ELLR matrix), pre-computed and stored with blob-ELLR (named blob-ELLR matrix) respectively. Figure 7 shows the memory requirement of the sparse data structure (i.e. ELLR and blob-ELLR on the GPU respectively). In general, the requirements rapidly grow with the dataset size increasing, approaching 3.5G in the large-sized dataset. This amount turns out to be a problem owing to the limited memory of GPUs. Since the limited memory is only 896 MB in GTX295, the weighted matrices using the ELLR format cannot be stored in the memory of the GPU. Similarly, the upper boundary imposed by the memory available in Tesla C2050 precludes addressing problem sizes requiring more than 3 GB of memory. However, in the blob-ELLR matrix structure, three symmetry relationships can greatly decrease the memory demands and make them affordable on GPUs.

Table 1 shows the computation times of different methods on the CPU Intel Xeon X5650, GTX295 and Tesla C2050s for the three datasets. All the computation times of 3D reconstruction are given for different number of iterations. As shown in Table 1, the running times of the blob-ELLR matrix method are less than those of the standard and ELLR matrix method both on CPU and GPUs. Due to the memory requirements of the medium-sized and large-sized data more than 896 MB, the ELLR matrix method cannot be used for these dataset on the GTX295. The ELLR matrix method cannot also be implemented for the large-sized data due to the limited 3 GB memory of the Tesla C2050s. But the problem can be addressed by adopting the blob-ELLR matrix method. These results demonstrate that the blob-ELLR matrix method succeeds in reducing the computation time of 3D-ET reconstruction.

**Table 1:** Running times (s)

datasets	iteration number	CPU					
		
		*standard*			*ELLR *		*blob-ELLR*
512 × 512	1	1413.12			235.52		204.12
	5	4025.39			685.64		586.37
	10	6498.18			1116.54		968.39
	25	16032.87			2788.84		2423.67

1024 × 1024	1	18708.48			3225.60		2763.23
	5	60328.71			10473.51		9126.34
	10	93052.42			16423.07		14228.64
	25	210824.49			37524.64		32787.49

2048 × 2048	1	111288.32			19476.48		17147.66
	5	253034.69			44543.49		39536.56
	10	429278.31			76382.34		68356.32
	25	1006392.84			182980.72		164443.73

**datasets**	**iteration number**	**GTX295**			**Tesla C2050**		
		
		***standard***	***ELLR***	***blob-ELLR***	***standard***	***ELLR***	***blob-ELLR***

512 × 512	1	14.63	7.88	5.76	8.02	6.34	3.95
	5	42.36	24.31	14.35	23.26	18.45	11.92
	10	66.36	47.89	30.32	55.03	39.04	28.41
	25	155.45	110.83	82.31	137.82	93.92	72.01

1024 × 1024	1	162.54	-	79.39	98.73	81.83	57.07
	5	523.13	-	289.43	329.31	265.81	194.29
	10	742.02	-	479.30	492.81	389.32	294.62
	25	1784.75	-	937.41	1092.71	867.92	687.05

2048 × 2048	1	869.29	-	481.09	502.42	-	337.64
	5	1954.38	-	1226.74	1192.51	-	811.03
	10	3476.94	-	2143.93	2076.93	-	1423.95
	25	7986.42	-	6105.83	4691.93	-	3374.52

**Figure 8:**
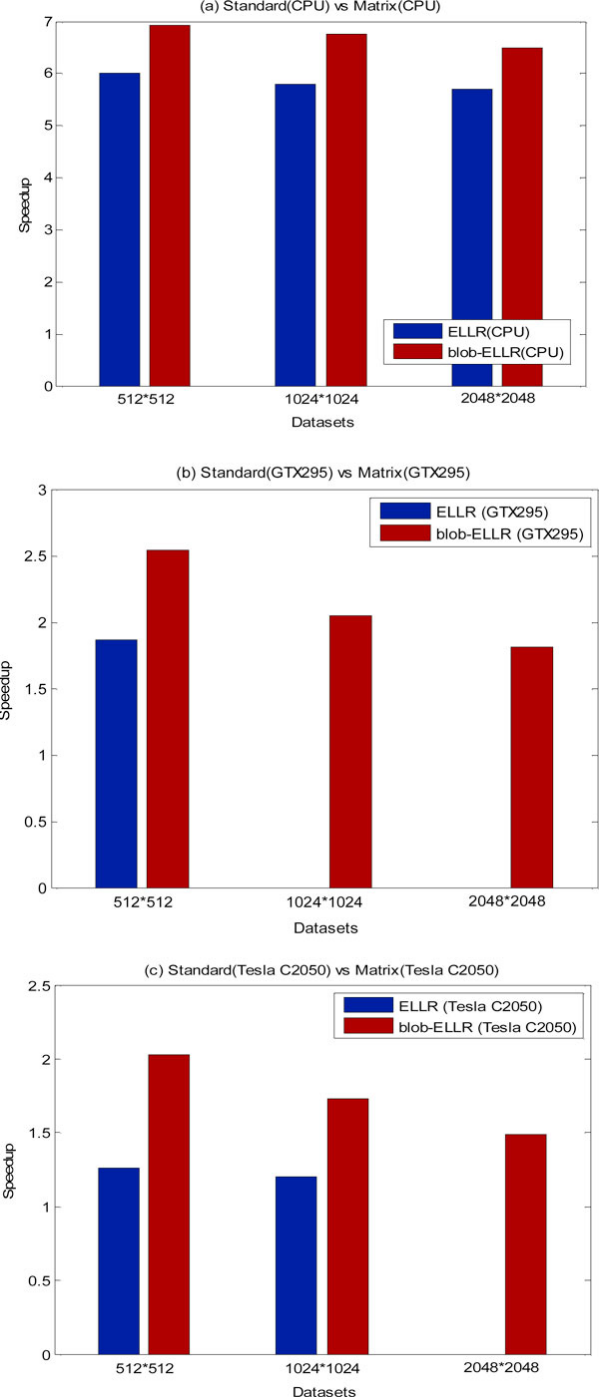
**Speedup factors of the different matrix methods (ELLR and blob-ELLR)**. The results are speedup factors for one iteration over the standard method on the CPU (a), the GTX295 (b) and the Tesla C2050s (c), respectively.

**Figure 9:**
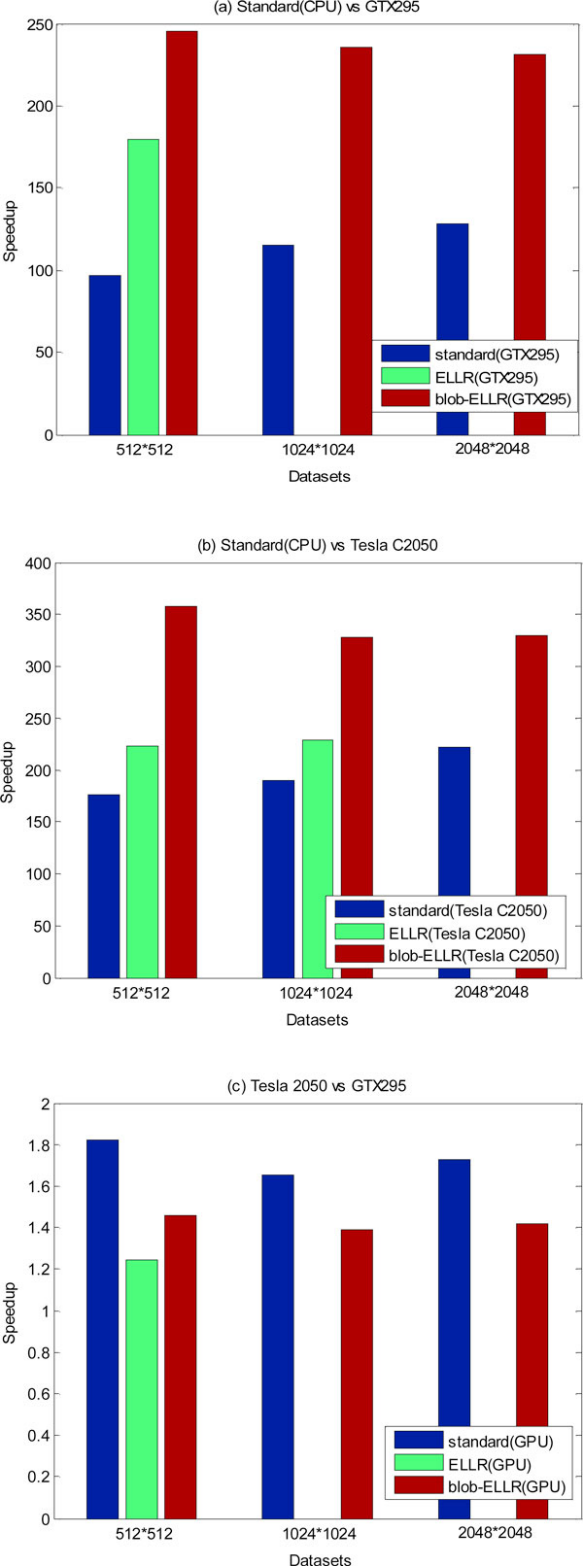
**Speedup factors derived from the different methods (standard, ELLR matrix and blob-ELLR matrix methods)**. The results are speedup factors for one iteration on the GTX295 vs. the standard method on the CPU (a), Tesla C2050 vs. the standard method on the CPU (b) and Tesla 2050 vs. GTX295 (c), respectively.

In order to estimate the performance of the matrix methods (i.e. ELLR matrix and blob-ELLR matrix) on the different platforms, the speedup factors against the standard method are showed in Figure 8. For the clear and brief description, we only show the results of the three datasets for one iteration. From Figure 8(a), we can see that the ELLR matrix method exhibits acceleration factors approaching to 6×, and the blob-ELLR matrix method obtains higher speedup factors almost 7× on the CPU. Figure 8(b) and 8(c) show a comparison of the ELLR matrix and blob-ELLR matrix methods for the GTX295 and Tesla C2050s. In Figure 8(b), due to the limited memory, the ELLR matrix method for both the medium-sized and large-sized data cannot be implemented on the GTX295. It is clearly seen that the blob-ELLR matrix method yields better speedups than the ELLR matrix method on the GTX295. Figure 8(c) shows the similar better performance of the blob-ELLR matrix method than that of the ELLR matrix method on the Tesla C2050s. Figure 9 compares the speedup factors of different methods on the GPU vs. the standard method on the CPU for one iteration. The blob-ELLR matrix method exhibits excellent acceleration factors compared with the other methods. As shown in Figure 9(a), the speedups of the standard method on the GTX295 vs. the CPU are almost 100× for three datasets. In the ELLR matrix method, the speedup is almost 160× for the first dataset. And in the case of the blob-ELLR matrix method, the acceleration factors increase and reach up to 200× for three datasets. The acceleration factors of the different methods on the Tesla C2050s over the standard method on the CPU are also presented in Figure 9(b). As shown in Figure 9(c), we directly calculated the speedups of Tesla 2050 vs. GTX295 for the different approaches: standard, ELLR and blob-ELLR in order to compare the performance of different GPU platforms. Since the computing ability of Tesla C2050 is better than that of GTX295, the speedups on the Tesla C2050s are larger than those on the GTX295 for all the three datasets. Those experiments would confirm the better performance of Tesla C2050 with respect to GTX295. From Figure 9, it is clear that the blob-ELLR matrix method can reduce the memory requirement of the weighted matrix and yield the best performance compared with the ELLR matrix and the standard methods on the two different GPU platforms.

## Conclusions

ET allows elucidation of the molecular architecture of complex biological specimens. Blob-based iterative methods yield better results than other methods, but are not used extensively in ET because of their huge computational demands. Multi-GPUs have emerged as powerful platforms to cope with the computational requirements, but have the difficulties due to the synchronous communication and limited memory of GPUs. In this work, we present a multilevel parallel strategy combined with an asynchronous communication scheme and a blob-ELLR data structure to perform high-performance blob-based iterative reconstruction in ET on multi-GPUs. The asynchronous communication scheme is used to minimize the idle GPU time. The blob-ELLR structure only needs nearly 1/16 of the storage space in comparison with the ELLR storage structure and yields significant acceleration compared to the standard and ELLR matrix methods. In this work, adopting the multilevel parallel strategy with the asynchronous communication scheme and the blob-ELLR data structure, we have performed the parallel 3D-ET reconstruction using SIRT on multi-GPUs. In fact, the parallel strategy proposed can be also easily applied to the other simultaneous methods, e.g. CAV. In the future work, we will further investigate and implement the multilevel parallel strategy and the asynchronous communication scheme on a many-GPU cluster.

## Competing interests

The authors declare that they have no competing interests.

## Authors' contributions

XHW carried out the design and implementation of the algorithms, and wrote the manuscript. FZ participated in the design and implementation of the algorithms and modified the manuscript. QC participated in the design of the design of the algorithms. ZYL participated in the design of the algorithms and modified the manuscript. All authors read and approved the final manuscript.
